# Supplementary material for: Development and validation of a clinical instrument to predict risk of an adverse drug reactions in hospitalized patients
Source: PLoS One. 2020 Dec 11;15(12):e0243714. doi: 10.1371/journal.pone.0243714 (PMC7732084; doi:10.1371/journal.pone.0243714)
Supplement: S1 Table — (DOCX) [file pone.0243714.s002.docx]

S1 Table.

Supplementary Table – Profile of adverse reactions identified and pharmacological classes involved.

| ADRs | Related drugs class | n | % |
| --- | --- | --- | --- |
| Hypoglycemia  (95 / 27.7%) | Insulin | 87 | 25,4 |
|  | Sulfonylureas | 5 | 1,4 |
|  | Biguanides | 3 | 0,9 |
| Hypotension  ( 68 / 19,8%) | ACE inhibitors | 26 | 7,6 |
|  | High-ceiling diuretics | 14 | 4,1 |
|  | Beta blocking agents | 9 | 2,6 |
|  | Direct vasodilators | 8 | 2,3 |
|  | Angiotensin II receptor blockers | 6 | 1,7 |
|  | Calcium channel blockers | 2 | 0,6 |
|  | Potassium-sparing agents | 2 | 0,6 |
|  | Others drugs | 2 | 0,6 |
| Nausea & Vomiting  (45 / 13.1%) | Opioid analgesic | 18 | 5,2 |
|  | General anesthesic | 4 | 1,2 |
|  | Laxatives | 4 | 1,2 |
|  | Antimycobacterials | 3 | 0,9 |
|  | Corticosteroid | 2 | 0,6 |
|  | Beta-lactams antibacterials | 2 | 0,6 |
|  | Others drugs | 13 | 3,6 |
| Hemorrhages or high INR  (33 / 9.6%) | Antithrombotic agent | 30 | 8,7 |
|  | Nonsteoidal anti-inflammatory | 3 | 0,9 |
| Hypokalemia (33 / 9.6%) | High-ceiling diuretics | 33 | 9,6 |
| Renal insufficiency  (21 / 6.1%) | ACE inhibitors | 5 | 1,4 |
|  | High-ceiling diuretics | 4 | 1,2 |
|  | Iodine contrast | 4 | 1,2 |
|  | Angiotensin II receptor blockers | 2 | 0,6 |
|  | Potassium-sparing agents | 2 | 0,6 |
|  | Glycopeptideantibacterials | 2 | 0,6 |
|  | Others drugs | 2 | 0,6 |
| Sedation & disorientation  (14 / 4.0%) | Benzodiazepine | 10 | 2,9 |
|  | Antipsychotic | 2 | 0,6 |
|  | Antihistamine | 2 | 0,6 |
| Hyperkalemia  (12 / 3.5%) | ACE inhibitors | 5 | 1,4 |
|  | Angiotensin II receptor blockers | 1 | 0,3 |
|  | Potassium-sparing agents | 5 | 1,4 |
| Others ADRs  (22 / 6.4%) | Others drugs | 21 | 6,1 |
| Total | | 343 | 100 |
