# Supplementary material for: Development and validation of a clinical instrument to predict risk of an adverse drug reactions in hospitalized patients
Source: PLoS One. 2020 Dec 11;15(12):e0243714. doi: 10.1371/journal.pone.0243714 (PMC7732084; doi:10.1371/journal.pone.0243714)
Supplement: Attachment — Submitted filename: Response_to_Reviewers_minorrev.docx [file pone.0243714.s004.docx]

**Response to Reviewers**

We are greatly indebted to the Editor by his careful review of the manuscript and the valuable suggestions. We accepted all the comments and made the appropriate changes in the manuscript, that we detail in this rebuttal letter to the academic editor.

**1. Title: The current title is unclear. The tool is not for "risk assessment of adverse reactions" but rather a tool to predict risk of an adverse reaction. Suggest revise to make this clearer, for example: Development and validation of a clinical instrument to predict risk of an adverse drug reaction in hospitalised patients.**

**Response:** We changed the title, as requested, from:

*“Development and validation of a clinical instrument for risk assessment of an adverse drug reactions in hospitalized patients”*

To:

*“Development and validation of a clinical instrument to predict risk of an adverse drug reactions in hospitalized patients”*

**2. There is no information included in the paper as to what ADRs were identified, which organ system was implicated, and what the implicated medicines were. Please can the authors include some description of the ADRs that were identified in the cases, and the medicines implicated in these adverse reactions. A supplementary table detailing the ADRs identified in the case, and implicated drugs. would be very useful.**

**Response:** We have added in the text (line 252, page 11) information about the most frequent adverse reactions and the medications involved. We prepared a table with all the reactions collected in the patient cases. The table was attached as a supplementary table and included in Supporting Information section (line 603, page 28).

*“Hypoglycemia related to insulin administration was the most common ADR (87, 25.4%), followed by hypotension (68, 19.8%) due to the use of different pharmacological classes of antihypertensive, highlighting the angiotensin-converting enzyme inhibitors (26, 7.6%) and loop diuretics (14, 4.1%). The occurrence of nausea and vomiting was observed in 13.1% (45) of the cases, related to several pharmacological classes such as opioid analgesics (18, 5.2%), general anesthetics (4, 1.2%) and laxatives (4, 1.2%). The other ADRs and medications involved can be seen in table 02.”*

***Supplementary Table – Profile of adverse reactions identified and pharmacological classes involved.***

| *ADRs* | *Related drugs class* | *n* | *%* |
| --- | --- | --- | --- |
| *Hypoglycemia*  *(95 / 27.7%)* | *Insulin* | *87* | *25,4* |
|  | *Sulfonylureas* | *5* | *1,4* |
|  | *Biguanides* | *3* | *0,9* |
| *Hypotension*  *( 68 / 19,8%)* | *ACE inhibitors* | *26* | *7,6* |
|  | *High-ceiling diuretics* | *14* | *4,1* |
|  | *Beta blocking agents* | *9* | *2,6* |
|  | *Direct vasodilators* | *8* | *2,3* |
|  | *Angiotensin II receptor blockers* | *6* | *1,7* |
|  | *Calcium channel blockers* | *2* | *0,6* |
|  | *Potassium-sparing agents* | *2* | *0,6* |
|  | *Others drugs* | *2* | *0,6* |
| *Nausea & Vomiting*  *(45 / 13.1%)* | *Opioid analgesic* | *18* | *5,2* |
|  | *General anesthesic* | *4* | *1,2* |
|  | *Laxatives* | *4* | *1,2* |
|  | *Antimycobacterials* | *3* | *0,9* |
|  | *Corticosteroid* | *2* | *0,6* |
|  | *Beta-lactams antibacterials* | *2* | *0,6* |
|  | *Others drugs* | *13* | *3,6* |
| *Hemorrhages or high INR*  *(33 / 9.6%)* | *Antithrombotic agent* | *30* | *8,7* |
|  | *Nonsteoidal anti-inflammatory* | *3* | *0,9* |
| *Hypokalemia (33 / 9.6%)* | *High-ceiling diuretics* | *33* | *9,6* |
| *Renal insufficiency*  *(21 / 6.1%)* | *ACE inhibitors* | *5* | *1,4* |
|  | *High-ceiling diuretics* | *4* | *1,2* |
|  | *Iodine contrast* | *4* | *1,2* |
|  | *Angiotensin II receptor blockers* | *2* | *0,6* |
|  | *Potassium-sparing agents* | *2* | *0,6* |
|  | *Glycopeptideantibacterials* | *2* | *0,6* |
|  | *Others drugs* | *2* | *0,6* |
| *Sedation & disorientation*  *(14 / 4.0%)* | *Benzodiazepine* | *10* | *2,9* |
|  | *Antipsychotic* | *2* | *0,6* |
|  | *Antihistamine* | *2* | *0,6* |
| *Hyperkalemia*  *(12 / 3.5%)* | *ACE inhibitors* | *5* | *1,4* |
|  | *Angiotensin II receptor blockers* | *1* | *0,3* |
|  | *Potassium-sparing agents* | *5* | *1,4* |
| *Others ADRs*  *(22 / 6.4%)* | *Others drugs* | *21* | *6,1* |
| *Total* | | *343* | *100* |

**3. Some comment on the prevalent ADRs in the cases should be included. This is important for assessment of generalizability and clinical utility of this instrument.**

**Response:** We included a paragraph in the discussion session (line 346, page 20) on the findings of the most frequent reactions and medications involved.

*“It is observed that almost all RAM are typical of the main drugs used, such as hypoglycemia and hypotension associated with hypoglycemic and antihypertensive drugs, respectively. Insulin is commonly related to adverse reactions like this in the hospital setting [22]. The drug-dependent dose reactions may occur due to limitations, such as the controlled diet with limited carbohydrates in the hospital environment and bed restriction [23], resulting in the particularity of the individual dose, subject to the need for constant dose adjustments [24, 25]. Hypotension also seems to be related to the patient's fragility, length of hospital stay and, mainly, age and concomitant use of more than one antihypertensive [26, 27].”*

**4. In response to a reviewer's comment on table 2, the following text was added to table 2 in this revision: “All variables were collected at the time of the ADR in the cases and of randomization in the controls.”
The controls were not randomized, so it is unclear what this refers to. Please correct this footnote.**

**Response:** The controls were randomized, as explained in Methods section, line 126, page 6: *“All patients with suspected ADR were included in the study as cases at the time of the event. The same day, two controls were randomly selected among all patients hospitalized in the eligible wards hospitalized within 5 days of the admission date of the case.”*.
